# CDC’s National Asthma Control Program: Looking Back with an Eye Toward the Future

**DOI:** 10.5888/pcd21.240051

**Published:** 2024-09-19

**Authors:** Alisha A. Etheredge, Carlene Graham, Maureen Wilce, Joy Hsu, Scott A. Damon, Josephine Malilay, Henry Falk, Kanta Sircar, Hailay Teklehaimanot, Erik R. Svendsen

**Affiliations:** 1 Centers for Disease Control and Prevention, National Center for Chronic Disease Prevention and Health Promotion, Division of Population Health, Healthy Aging Branch, Atlanta, Georgia; 2 Centers for Disease Control and Prevention, National Center for Environmental Health, Division of Environmental Health Science and Practice, Asthma and Air Quality Branch, Atlanta, Georgia; 3 Centers for Disease Control and Prevention, National Center for Environmental Health, Atlanta, Georgia; 4 Centers for Disease Control and Prevention, National Center for Environmental Health, Division of Environmental Health Science and Practice, Atlanta, Georgia

## Introduction

Asthma is one of the most prevalent chronic respiratory diseases among adults and the most common chronic disease among children in the US, costing the nation more than $80 billion each year (1). More than 25 years ago, the increasing prevalence of asthma prompted the Centers for Disease Control and Prevention (CDC) to establish a nationwide program to address asthma’s rising public health burden. When the National Asthma Control Program (NACP) began in 1999, early efforts focused on capacity building of states, territories, and local levels; tracking the burden of asthma by collecting and analyzing surveillance data; identifying and implementing science-based interventions to help individuals manage and control their asthma; and establishing and maintaining national, state, and community partnerships to reduce asthma burden with a focus on states with high burdens. To date, the NACP has played a critical role in efforts to help millions of people in the US with asthma. Data from the past 10 years indicate asthma prevalence has decreased among some populations; however, racial and ethnic disparities persist (2). We provide a brief history of the NACP, from its origins to the rich successes and challenges of building an environmental public health program. This essay is intended to raise awareness and support for state and local public health asthma control efforts, and lessons learned through the NACP’s history and current state can assist others in planning environmental public health programs in asthma and other chronic and environmental health topics to achieve population-level impact.

## Where We Started

The NACP originated in CDC’s then-named Air Pollution and Respiratory Health Branch (APRHB), which was created shortly after the adoption of the Clean Air Act amendments in 1990. The National Institutes of Health’s National Heart, Lung, and Blood Institute introduced the first national evidence-based guidelines for diagnosing and managing asthma in 1991 (3). The guidelines aimed to help health care professionals bridge the gap between current knowledge and practice. The President’s Task Force on Environmental Health Risks and Safety Risks to Children in the late 1990s prioritized children’s environmental health, with specific emphasis on childhood asthma.

In 1997, CDC, the American Association of Health Plans and Prevention, and Emory University collaborated with diverse organizations to form a coalition addressing asthma in 400 children from low-income households in Atlanta’s economic empowerment zone. The economic empowerment zone is an area of economically disadvantaged urban communities receiving supports such as financial incentives, tax benefits, grants, technical assistance, and access to resources. The coalition implemented the ZAP Asthma Program (https://stacks.cdc.gov/view/cdc/41511) as a community-based initiative by using trained community health workers (CHWs). CHWs conducted asthma home visits, educated parents and caregivers about environmental triggers, and implemented interventions, including in-home environmental assessments and asthma self-management education. This program demonstrated the value of engaging communities to develop and implement asthma control interventions that fit their local context.

## Tracking the Burden of Asthma

In 1998, CDC published the first national review of asthma surveillance data, revealing an increase in asthma prevalence and death rates in the US from 1960 through 1995 (4). However, the available surveillance data were insufficient to assess state and local trends. In 1999, Congress appropriated funding to CDC to conduct local asthma surveillance activities and compile and annually publish data on the prevalence of children having asthma in each state as well as the childhood asthma death rate nationally and in each state. Recognizing the need for a more comprehensive approach, CDC began developing the NACP, initiating a surveillance cooperative agreement program in 1999. The program aimed to establish and evaluate a sentinel surveillance system in hospital emergency departments, focusing on monitoring trends and identifying reasons for receiving asthma care. This proactive step was driven by the understanding that enhanced state and local asthma surveillance could lead to more effective prevention and management strategies. The 1998 national asthma surveillance report significantly raised awareness of asthma’s burden in the US and is cited over 945 times in peer-reviewed literature (4).

Over the years, the NACP played a pivotal role in tracking asthma burden through surveillance and epidemiology. Key initiatives include integration of asthma modules within the Behavioral Risk Factor Surveillance System (BRFSS), the first effort to systematically collect state-based asthma prevalence data. In 2003, NACP developed and tested a National Asthma Survey (NAS) through the State and Local Area Integrated Telephone Survey (SLAITS), later renamed the Asthma Call-back Survey (ACBS), which, by 2010, expanded to include 40 states, the District of Columbia, and Puerto Rico. The survey collected household and health data monthly from selected respondents, allowing NACP to investigate the health, socioeconomic, behavioral, and environmental predictors related to better asthma control. ACBS was a cornerstone for understanding asthma prevalence, symptoms, health care use, and environmental risk factors. These comprehensive data collection efforts, due in part to NACP investment in resources and design of survey questions, significantly enhanced public health decision-making, enabling focused interventions and evaluations to reduce the burden of asthma at both the state and local levels.

## Program Interventions

NACP has dedicated substantial funding and technical assistance to partners to develop interventions, scientifically evaluate their effectiveness, and translate them for widespread implementation across diverse communities. In 2004, NACP funded 7 cities through the Controlling Asthma in American Cities Project (CAACP), aiming to translate scientific advances in the treatment of asthma into innovative, comprehensive approaches for improving asthma control among children who are up to 18 years old living in economically disadvantaged urban communities with a high asthma burden (5). Some of the interventions tailored to suit the specific circumstances of the local communities included educating day care providers and parents on asthma management for young children, integrating asthma self-management training into faith-based organizations, establishing links between high-risk children and specialty asthma services through schools, training community pharmacists to educate individuals with asthma on proper medication usage, and collaborating with managed care plans to ensure reimbursement for asthma self-management training.

In 2009, leveraging insights from these past projects, CDC allocated funding for health departments to establish asthma programs through the Addressing Asthma from a Public Health Perspective cooperative agreement. This effort supported 34 states, the District of Columbia, and Puerto Rico, fostering collaborative efforts to implement evidence-based interventions, enhance asthma surveillance, and develop and implement state asthma plans with their state and local partners over a 5-year period. Valuable lessons include promoting cross-jurisdictional collaboration among funded health departments to enhance asthma surveillance and program activities, establishing mentoring programs for newly funded health departments to facilitate knowledge exchange and capacity building, and fostering creativity within partnerships to facilitate innovative strategies and sustainability. Subsequent NACP-supported cooperative agreements were launched in 2014, 2016, and 2019, with each iteration leveraging successes and incorporating lessons learned from the previous project periods. As of August 2024, NACP funds 23 state, 1 territorial, and 1 local public health departments.

## Evaluation Approach

In the 2009 funding cycle, the program expanded evaluation by mandating that funded state asthma programs allocate a half-time staff person to support evaluation activities. Programs worked with partners in the first 6 months of the cooperative agreement to develop an evaluation agenda, referred to as a strategic evaluation plan. These plans ensured that evaluations were responsive to broad information needs and conducted in a coherent sequence; program planning was strengthened through strategic evaluative thinking. Programs developed individual evaluation plans to guide evaluations of major program components: partnerships, surveillance, and interventions. To support these activities, NACP established a team of evaluation technical advisors and created “Learning and Growing through Evaluation,” a series of evaluation guides based on the CDC Framework for Program Evaluation in Public Health (6). These resources and a suite of other evaluation tools facilitated hundreds of evaluations. For example, since 2014, the Utah Asthma Program and the Utah Pediatric Partnership to Improve Healthcare Quality have collaborated with 37 clinics to improve primary care diagnosis and team-based management of asthma patients through a 6-month learning collaborative. A follow-up evaluation conducted in 2022 demonstrated significant improvements in various areas among participating clinics: for example, an increase in the use of a standardized asthma assessment tool from 38% to 90% and an increase in patients with an active asthma action plan or self-management plan on file from 55% to 100%. Although later funding cycles dropped the staffing requirement, evaluation remains integral, with programs using findings to expand partnerships, create new surveillance products, and make programming decisions that increased efficiency and effectiveness. NACP’s efforts have been acknowledged in the evaluation field, culminating in the creation of an evaluation textbook, *Planting the Seeds for High-Quality Program Evaluation in Public Health*, in collaboration with partners in 2021 (7).

## Developing a Framework for Asthma Programs

In 2007, NACP initiated a comprehensive review of asthma interventions through the Community Preventive Services Task Force (8). Findings from the Task Force solidified NACP’s support of community-level implementation of multicomponent interventions to address asthma, including guidelines-based medical management, asthma self-management education (AS-ME), indoor and outdoor trigger reduction interventions, and linkages to services to help reduce exposure to asthma triggers (8). Interventions that use policy, systems, and environmental approaches at the population level can help expand the reach of public health efforts to control and manage asthma. In 2018, NACP formally characterized this multistrategy approach in a technical package known as EXHALE, designed to inform decision-making for communities, organizations, and states as well as facilitate multisector collaborations that would build on asthma-related public health and health care collaboration in CDC’s 6|18 initiative (9,10). EXHALE is a set of 6 strategies used to facilitate asthma control in children and adults. Each strategy is designed to reduce emergency department visits and hospitalizations, which are key indicators of poor asthma control for individuals. The strategies also improve health equity by encouraging public health interventions that directly affect health inequities, such as connecting people with asthma to local support services to improve conditions where people live, work, play, and learn. Since the development of the 2019 asthma cooperative agreement, NACP has applied the EXHALE technical package as a framework for public health asthma program development. Given evidence that a multicomponent approach to controlling asthma is more effective than individual strategies applied in isolation (9), multiple federal agencies have used EXHALE in their asthma-related activities, including the Centers for Medicare and Medicaid Services and the Indian Health Service.

## Establishing and Maintaining Partnerships

Over the past 22 years, NACP has partnered with nongovernmental organizations to promote intervention programs and expand outreach across groups from diverse racial, ethnic, and socioeconomic backgrounds to implement activities such as asthma health education enhancement programs. These organizations — American Lung Association, Allergy & Asthma Network, and Asthma and Allergy Foundation of America (the National Environmental Education Foundation was added in 2010) — have worked to promote evidence-based asthma strategies through patient-oriented and clinician-oriented education and multisector partnerships.

## The Future Direction of NACP

The NACP has made advancements over the past 25 years in developing and implementing a public health approach to address asthma. Substandard housing, particularly in urban and rural neighborhoods, and racial and ethnic disparities are associated with poor asthma outcomes (11–13). These factors contribute to increased exposure to environmental triggers, such as pollutants and allergens, and inadequate access to health care services (11,12,14). Addressing social determinants of health (SDOH) is key to reducing asthma disparities and requires a multifaceted approach (9,14). Two strategies highlighted within NACP’s EXHALE technical package can address SDOH: 1) linkages and coordination of care across settings, which can be advanced by implementing Medicaid health homes and patient-centered medical homes, integrating community health workers and case managers to facilitate resource linkage and community referrals and provide asthma self-management and education training, and creating school-based programs that provide coordinated care through school nurses or other staff; and 2) environmental policies or best practices to reduce asthma triggers from indoor, outdoor, and occupational sources, which can be advanced by facilitating smokefree policies and clean diesel school buses, eliminating exposure to asthma triggers in schools, and facilitating home energy efficiency (including home weatherization assistance programs).

Expanding implementation of the evidence-based strategies presented in the EXHALE technical package provides potential to address SDOH, but these strategies cannot be carried out by public health alone. Partnerships are integral vehicles for driving EXHALE implementation, and collaborating across multiple sectors such as schools, health care systems, housing organizations, transportation organizations, community and faith-based organizations, and tribal communities can lead to comprehensive solutions that address the multifaceted causes of asthma disparities. Strategies to build and sustain these partnerships include identifying common goals and vision, leveraging existing networks such as coalitions, identifying partnership champions, having strong leadership, and having clear structures and processes. By working together across sectors, resources can be pooled effectively to achieve better outcomes.

NACP aims to expand its reach to more states with incremental approaches to strengthen their asthma programs. This could be a challenge given the current level of funding for NACP; however, all states would benefit from receiving the support and resources needed to increase implementation of EXHALE strategies for asthma, particularly in communities that have been marginalized. If funding remains the same, NACP will strategically maximize available resources through focused investments on selected cost-efficient, evidence-based, EXHALE-related public health activities, especially in communities at higher risk of asthma-related emergencies (eg, activities to improve sustainability of results-based health equity partnerships to reduce asthma-related illness, death, and disparities). NACP will also work to further identify, strengthen, and leverage existing asthma surveillance systems to inform public health policy and planning while also exploring use of innovative strategies that address environmental risk factors affecting millions of people in the US with asthma, including poor air quality and wildfires.
